# Gut Microbiota, Blood Metabolites, and Spleen Immunity in Broiler Chickens Fed Berry Pomaces and Phenolic-Enriched Extractives

**DOI:** 10.3389/fvets.2020.00150

**Published:** 2020-04-22

**Authors:** Quail Das, Md. Rashedul Islam, Dion Lepp, Joshua Tang, Xianhua Yin, Lili Mats, Huaizhi Liu, Kelly Ross, Yan Martel Kennes, Hassina Yacini, Keith Warriner, Massimo F. Marcone, Moussa S. Diarra

**Affiliations:** ^1^Department of Food Science, University of Guelph, Guelph, ON, Canada; ^2^Guelph Research and Development Centre, Agriculture and Agri-Food Canada, Guelph, ON, Canada; ^3^Summerland Research and Development Centre, Agriculture and Agri-Food Canada, Summerland, BC, Canada; ^4^Centre de Recherche en Sciences Animales de Deschambault, Deschambault, QC, Canada

**Keywords:** broilers, cranberry and blueberry pomaces, blood metabolites, gut microbiota, spleen, immunity

## Abstract

This study evaluated the performance, gut microbiota, and blood metabolites in broiler chickens fed cranberry and blueberry products for 30 days. A total of 2,800 male day-old broiler Cobb-500 chicks were randomly distributed between 10 diets: control basal diet; basal diet with bacitracin (BACI); four basal diets with 1 and 2% of cranberry (CP1, CP2) and blueberry (BP1, BP2) pomaces; and four basal diets supplemented with ethanolic extracts of cranberry (COH150, COH300) or blueberry (BOH150, BOH300) pomaces. All groups were composed of seven replicates (40 birds per replicate). Cecal and cloacal samples were collected for bacterial counts and 16S rRNA gene sequencing. Blood samples and spleens were analyzed for blood metabolites and gene expressions, respectively. The supplementation of COH300 and BOH300 significantly increased the body weight (BW) during the starting and growing phases, respectively, while COH150 improved (*P* < 0.05) the overall cumulated feed efficiency (FE) compared to control. The lowest prevalence (*P* = 0.01) of necrotic enteritis was observed with CP1 and BP1 compared to BACI and control. Cranberry pomace significantly increased the quinic acid level in blood plasma compared to other treatments. At days 21 and 28 of age, the lowest (*P* < 0.05) levels of triglyceride and alanine aminotransferase were observed in cranberry pomace and blueberry product–fed birds, respectively suggesting that berry feeding influenced the lipid metabolism and serum enzyme levels. The highest relative abundance of *Lactobacillaceae* was found in ceca of birds fed CP2 (*P* < 0.05). In the cloaca, BOH300 significantly (*P* < 0.005) increased the abundances of *Acidobacteria* and *Lactobacillaceae*. *Actinobacteria* showed a significant (*P* < 0.05) negative correlation with feed intake (FI) and FE in COH300-treated birds, whereas *Proteobacteria* positively correlated with the BW but negatively correlated with FI and FE, during the growing phase. In the spleen, cranberry products did not induce the release of any pro-inflammatory cytokines but upregulated the expression of several genes (IL4, IL5, CSF2, and HMBS) involved in adaptive immune responses in broilers. This study demonstrated that feed supplementation with berry products could promote the intestinal health by modulating the dynamics of the gut microbiota while influencing the metabolism in broilers.

## Introduction

Necrotic enteritis (NE) caused by *Clostridium perfringens* and coccidiosis induced by *Eimeria* spp. are intestinal diseases that cause important economic losses to poultry production due to productivity losses, cost of treatments, and premature deaths (1). In conventional broiler production, antimicrobials such as bacitracin (BACI) and salinomycin are used in feed to prevent such intestinal diseases, resulting in an improvement of feed conversion and body weight (BW) gain. Antibiotic-free (raised without antibiotic: RWA) and organic poultry production are increasing in developed countries in response to consumers' demand for non-conventionally produced food, driven by issues including antimicrobial resistance (AMR) (2). Such production requires no use of traditional antibiotics in intensive production and/or free-range systems (outdoor access to pasture) in organic production. Accordingly, the Chicken Farmers of Canada (CFC) recently decided to eliminate the preventive use of Category II antibiotics in 2018 and of Category III antibiotics by the end of 2020 (https://www.chickenfarmers.ca/antibiotics). However, RWA and organic (antibiotic-free) production systems in some countries appear to increase the exposure to environmental pathogenic bacteria such as *C. perfringens* (3), avian pathogenic *Escherichia coli:* APEC (4), *Campylobacter* spp., and *Salmonella enterica* serovars (5) that pose a threat to birds' health and food safety. Antibiotic-free poultry production systems were reportedly associated with poorer feed efficiency (FE), reduced weight gain, and BW at slaughter, along with an increased incidence of clinical and subclinical NE cases (6). Thus, efficient and cost-effective methods for maintaining/improving birds' health, reducing food safety risks (foodborne pathogens), and lessening negative environmental impacts of production are warranted for antibiotic-free poultry production.

Feed additives have received much attention since the ban of in-feed antibiotics as growth promoters in Europe in 2006 and recent restriction of their use in North America (7). Feed additives may have a pleiotropic effect on poultry and are used to increase palatability, improve nutrient availability, reduce endogenous protein production and losses, reduce pathogenic microbial growth, reduce inflammation and gut permeability, increase binding of toxins, enhance intestinal recovery and function, increase colonization, and improve microbiota balance (8). The gut microbiota in chicken plays an important role in maintaining overall health and in the development of the immune system and intestinal homeostasis, and provides protection against pathogens (9). However, environmental conditions (such as housing, feed access, etc.) and host factors (line, sex, age, and disease conditions) significantly influence the composition of the intestinal microbiota (10).

The use of fruit pomaces in animal production is gaining popularity (11). The North American cranberry (*Vaccinium macrocarpon*) and wild blueberry (*Vaccinium angustifolium*) are characterized by their high phenolic acids, proanthocyanidins, anthocyanins, flavonoids, and other insoluble fiber contents. Bioactive compounds from berry pomaces and their extracts exhibited a wide range of biological activities, including antioxidant, anti-carcinogenic, anti-inflammatory, anti-neurodegenerative, and antiviral (12, 13). These products showed concentration-dependent effects by modulating gut microbiota (14, 15). Therefore, it is appropriate to explore these berry by-products as resources for different value-added applications.

Although feeding practices are known to impact animal health and productivity, very limited research has been done on the effects of berry by-products as feed supplements on gut microbiota composition and blood metabolites in broiler chickens. We previously reported the potential of berry products in food production including feeding strategies to modulate gut microbiota in food animals (15, 16), and demonstrated that cranberry products enhanced immuno-defense mechanisms of chickens against infections (17). Moreover, cranberry pomace fractions were reported to inhibit growth of AMR *Salmonella* serovars while affecting the metabolism and nutrient uptake as well as expression of virulence factors in *Salmonella* Enteritidis from broilers (18). These studies imply that both cranberry and blueberry products could be developed to maintain or improve poultry productivity and safety. In the present study, we evaluated the growth performance, intestinal health, as well as cecal and cloacal microbiota in broiler chickens receiving organic cranberry and wild blueberry pomace and their phenolic-enriched extractives (ethanolic extracts) in feed. The impact of berry product feeding on blood metabolites was estimated during growing and finishing periods. In addition, correlations between abundances of cecal bacterial taxa, performance parameters, and blood metabolite profiles were determined. Furthermore, we investigated the gene profile of the spleen to get insight into the potential immune response of broilers to dietary cranberry products, for which limited data exist.

## Materials and Methods

### Animal Ethics

All experimental procedures performed in this study were approved (protocol #16-AV-314) by the Animal Care Committee of the Center de recherche en sciences animales de Deschambault (CRSAD, Deschambault, QC, Canada) according to guidelines described by the Canadian Council on Animal Care (19).

### Berry Products

Organic cranberry (CP: *V. macrocarpon*) and wild blueberry (BP: *V. angustifolium*) pomaces were prepared and characterized previously (13). Phenolic-rich pomaces were extracted with 80% ethanol from the CP and BP. After extraction, ethanol was removed from the CP and BP with a rotary evaporator and freeze-dried at −30°C to generate crude pomace extracts (COH and BOH) that were kept at −20°C until their use. Composition of the studied products including content in phenolic acids, tartaric esters, flavonols, anthocyanins, tannins, carbohydrate, lipids, proteins, and minerals such as Ca, Mg, Fe, Mn, and Cu has been previously reported (13).

### Broiler Chickens and Housing

A total of 2,800 male day-old broiler Cobb-500 chicks were randomly distributed between 70 floor pens (40 birds/pen) at the CRSAD (Deschambault, QC, Canada). Before placement, all chicks were visually examined for health, and inferior chicks were not included in the trial. The concrete floor was covered with ~3 in (7.6 cm) of clean softwood wood chips, and ventilation was provided by negative pressure with fans. Heat was provided by gas-fired brooders; water and feed were offered *ad libitum* through nipple drinkers and tube feeders, respectively. Birds were managed according to the Cobb recommendations (Cobb Breeder Management Guide and Vantress.com). The composition of the starter (days 0–10), grower (days 10–20), and finisher (days 20–30) diets included corn as the principal cereal, and soya and soybean cake as protein concentrates to meet the nutrient requirements for broiler Cobb-500 (20, 21).

### Study Design

The 70 pens were assigned to 10 treatments (7 pens/treatment) using a complete randomized block design. The 10 treatments consisted of: control negative (CON: non-medicated basal feed); basal feed supplemented with BACI (55 ppm); two groups receiving basal feed supplemented with 1 and 2% cranberry pomace (CP1 and CP2); two groups receiving basal feed fortified with 1 and 2% blueberry pomace (BP1 and BP2); two groups receiving basal feed supplemented with 150 and 300 ppm of cranberry ethanolic extracts (COH150 and COH300); and two groups receiving basal feed supplemented with 150 and 300 ppm of blueberry ethanolic extracts (BOH150 and BOH300). All birds were vaccinated against coccidiosis. The tested products were applied from day 0 until day 30 of age. No additional anticoccidials or antibiotics were administrated to the birds throughout the trial.

### Data Collection

Chicks were weighed at the start of the trial (day 0) and every week thereafter. Performance parameters including BW, feed intake (FI), and FE were measured at days 10 (phase 1), 20 (phase 2), and 30 (phase 3) from each pen (20). Birds were inspected at least twice daily. Any mortalities or culls were removed. The dates of removal and bird weights were recorded on a data capture sheet. Necropsies were performed by Services Vétérinaires Ambulatoires Triple-V Inc. (Acton Vale, QC, Canada) on all mortalities to determine the causes of death. Any birds showing signs of illness or distress were removed and humanely killed. During flock inspections, birds were observed for activities, and feed and water were checked to assure that each was always available.

### Sample Collection, Bacteriology, and Necropsy

At days 21 and 28, two birds/pen (seven pens/treatment) were randomly chosen and weighed individually. Blood samples were collected from wing veins, and then birds were sacrificed by cervical dislocation. Cecal contents and cloacal (fecal) samples were aseptically collected from each bird and transferred to sterile Whirl-Pak plastic bags (Nasco, Fort Atkinson, WI) and test tubes, respectively; immediately frozen (−20°C); and transported to the laboratory for microbiota analysis. The collected cecal samples were analyzed using culture methods on selective media: *C. perfringens* on cycloserine supplemented tryptose sulfite cycloserine (TSC) agar media, *E. coli* on CHROMagar™, and *Lactobacillus* on MRS agar media. The results of cecal microbial enumerations were log transformed before statistical analysis. Necropsy and scoring of intestinal lesions due to coccidiosis (*Eimeria acervulina, Eimeria maxima*, and *Eimeria tenella)* and NE due to *C. perfringens* were performed on all sacrificed birds by Services Vétérinaires Ambulatoires Triple-V Inc. Intestines were longitudinally opened to score mucosa for NE lesions for each of the upper and lower gut (including ceca) as well as for coccidiosis as previously described (20). Birds were also monitored for yolk sac infection (omphalitis), trachea integrity, pododermatitis, gizzard ulceration, intestinal tonus, airsacculitis, metatarsal, femoral head necrosis, and bursal size.

### Blood Serum Metabolites

Blood samples collected from birds on days 21 and 28 were allowed to clot at room temperature before centrifugation at 2,000 × g for 10 min for serum collection (15). Collected sera were transferred to sterile Eppendorf tubes and stored at −80°C until further analysis. Blood serum samples were assessed for 19 blood biochemistry parameters at the Animal Health Laboratory (University of Guelph, Guelph, ON, Canada) for: (1) enzymes: alanine aminotransferase (ALT), aspartate aminotransferase (AST), alkaline phosphatase (ALP), amylase (AMY), lipase (LIP), and gamma-glutamyltransferase (GGT); (2) minerals: calcium (Ca), iron (Fe), magnesium (Mg), and phosphorus (P); (3) glucose, lipids, cholesterol (CHO), high-density lipoprotein cholesterol (HDLC), triglyceride (TRIG), and non-esterified fatty acids (NEFA); and (4) protein: total proteins (TP), albumin (ALB), globulin (GLO), and ALB–GLO ratio (AGR).

### Phenolics in Blood Plasma by Liquid Chromatography–Mass Spectrometry

Individual blood samples from birds selected on day 21 were immediately centrifuged at 3,000 × g for 15 min at 4°C, and the plasma were collected and stored at −20°C. Chicken plasma samples were transferred to Eppendorf microcentrifuge tubes, mixed with 4°C cold acetonitrile 1:4 (v:v), and centrifuged at 12,000 × g at 4°C for 15 min to remove precipitates; supernatant was transferred to a pre-balanced 33 μm polymer reverse phase 96-well-plate (60 mg/well) to remove residual proteins. The filtrates were analyzed by LC-MS/MS using a Thermo Scientific™ Q-Exactive™ Orbitrap mass spectrometer equipped with a Vanquish™ Flex Binary UPLC System (Waltham, MA, USA). Data were acquired using Thermo Scientific™ Xcalibur™ 4.2 software and Thermo Scientific™ Standard Integration Software (SII). The chromatographic separation was performed on a ZORBAX RRHD Eclipse Plus Phenyl-Hexyl HPLC column (2.1 × 150 mm, 1.8 μm, Agilent, Mississauga, ON, Canada). The binary mobile phase consisted of solvent A (99.9% H_2_O/0.1% formic acid) and solvent B (99.9% ACN/0.1% formic acid). The following solvent gradient was used: 0–8 min, 0–24% B; 8–10 min, 24% B; 10–14 min, 24–60% B; 14–15 min, 60–100% B; 15–18 min, 100% B; 18–19 min, 100–0% B; 19–27 min, 0% B. The column compartment temperature was held at 40°C, the flow rate was set at 0.3 ml min^−1^, injection volume was set at 2 μl, and peaks were monitored at 280, 320, 360, and 520 nm. Mass spectrometry data were collected in negative ionization mode using the Full-MS/ddMS^2^ (TopN = 10) method, with NCE set at 30 and intensity threshold set at 1.0 e^5^ counts.

Data were analyzed and visualized using Thermo FreeStyle™ 1.5 software. Automated sample analysis was performed using Compound Discoverer 2.0 software. A modified template, “Untargeted food research workflow with statistics,” was used to perform sample grouping, peak detection, identification of unknowns, and differential analysis. The identification of compound in plasma was based on elemental composition prediction and subsequent ChemSpider database search (FullMS) as well as spectral matching of MS/MS data with the mzCloud library (MS^2^). Statistical analysis performed on detected peaks included differential analysis where *P*-values and fold changes were visualized using Volcano plots; a principal component analysis (PCA) plot was also generated by Compound Discoverer™ software.

### DNA Isolation for Microbiota Analysis

Genomic DNA for 16S rRNA sequencing was extracted from a cecal and cloacal sample using a QIAamp DNA Stool Mini Kit (Qiagen, Venlo, Netherlands) according to the manufacturer's instruction. DNA quality was checked by running on 1.0% agarose gel electrophoresis. DNA quantitation was performed using the Qubit^®^ 2.0 Fluorometer (Life Technologies, Carlsbad, CA, USA) and the Qubit dsDNA HS assay kit (Life Technologies, Carlsbad, CA, USA). Sequencing libraries of the 16S rRNA gene were prepared according to the Illumina 16S Metagenomic Sequencing Library Preparation Guide. Briefly, the 16S V3–V4 hypervariable region was amplified using primers (5′-CCTACGGGNGGCWGCAG-3′) and Bakt_805R (5′-GACTACHVGGGTATCTAATCC-3′) containing Illumina overhang adapter sequences (5′-TCGTCGGCAGCGTCAGATGTGTATAAGAGACAG and 5′-GTCTCGTGGGCTCGGAGATGTGTATAAGAGACAG, respectively) with KAPA HiFi HotStart ReadyMix (VWR), and purified with AMPure XP beads (Beckman Coulter). Sequencing adapters containing 8 bp indices were added to the 3′ and 5′ ends by PCR using the Nextera XT Index kit (Illumina) followed by a second purification with Ampure XP beads. Amplicons were quantified using the Quant-iT PicoGreen double-stranded DNA assay (Invitrogen), and equimolar ratios were pooled and combined with 10% equimolar PhiX DNA (Illumina) for sequencing on a MiSeq instrument, using the 600-cycle v3 kit (Illumina).

The data were analyzed by Quantitative Insights Into Microbial Ecology QIIME (version 1.9.1) (22). Paired-end reads (300 bp) were joined with fastq-join (23), and quality filtered and demultiplexed in QIIME using default settings. The reads were clustered at 97% sequence identity with UCLUST (24), and representative operational taxonomy units (OTUs) were picked using an open-reference approach (25). For both steps, the Greengenes representative OTU sequences (gg_otus_13_8), clustered at 97% identity, were used as reference. Taxa that could not be assigned a genus were presented as “unclassified” using the highest taxonomic level that could be assigned to them. The sequences were aligned against the Greengenes core set with PyNast (22), and a phylogenetic tree was constructed with FastTree (26). Alpha-diversity (within group) metrics were then calculated by QIIME, and a β-diversity (between group) distance matrix based on the unweighted UniFrac metric (27) was calculated, which was used for principal co-ordinate analysis (PCoA).

### Spleen RNA Extraction

At day 21 of age, spleens from sacrificed birds were collected and placed in tubes containing an RNA stabilization solution (AM7021, ThermoFisher Scientific) before being frozen. Three spleen samples for every six treatments (control, BACI, CP1, CP2, COH150, and COH300) were defrosted at room temperature for RNA extraction. Total RNA was prepared using the RNeasy® Mini Kit (Cat. No./ID: 74104 Qiagen) according to the manufacturer's instructions. Briefly, 10–15 mg of spleen samples were cut with sterile forceps and surgical blades and transferred into 600 μl RLT buffer with 1% beta-mercaptoethanol (Fisher Scientific). Cells were homogenized using a Pro 200 homogenizer (Pro Scientific). The homogenates were centrifuged for 3 min at 13,000 rpm, and the supernatants were transferred to microcentrifuge tubes followed by the addition of 70% ethanol. RNA was eluted using RNeasy Mini column (Cat. No./ID: 74104 Qiagen). The RNA quality was checked on an agarose gel, and the quantity and purity were measured with a Nanodrop spectrophotometer (260 and 260/280 nm respectively). The absence of genomic contamination was confirmed by running RNA samples with the GAPDH housekeeping gene using real-time PCR.

### Real-Time PCR

cDNA synthesis was performed using the Qiagen RT2 Profiler PCR Array Handbook 11/2018 according to manufacturer's instructions. In brief, 2 μg of total RNA of each sample was synthesized using RT2 First Strand Kit (Qiagen, Valencia, CA) and kept at −20°C until needed. cDNA samples were mixed with molecular-grade water and RT^2^ SYBR Green ROX qPCR Mastermix (Qiagen, Valencia, CA) according to manufacturer protocols and added to each well of the 96-well-plate purchased from the Chicken Innate & Adaptive Immune Response PCR Array (PAGG-052ZA, Qiagen). These plates were used to profile the expression of 84 genes involved in innate and adaptive immune response pathways. Gene expression was normalized using the housekeeping gene *ACTB* and *RPL4* selected by GeNorm assessment (28). Real-time PCR was performed using an Applied Biosystems 7500 Real-time PCR System with 7500 Software v2.3. Fold change in gene expression between the control and the remaining five treatments (BACI, CP1, CP2, COH150, and COH300) was calculated using the 2–ΔΔCt method, and *P*-value was calculated based on a Student's *t*-test between control and treatments at the significance level of 0.05.

### Statistical Analysis

Statistical analyses on growth performance, relative abundance of bacteria taxa, and severity of intestinal lesions (scores) were conducted according to a randomized complete block design using the General Linear Mixed Model (GLMM) procedure of the Statistical Analysis System version 9.4 (SAS Institute Inc., 2016, Cary, North Carolina, United State) (29). Treatments and sample sources (ceca and cloaca) were used as sources of variation and the individual pens as experimental units (seven pens/treatment group). Relationship between performance parameters, blood metabolites, and microbial taxa were estimated by non-parametric correlation measurements. Least significance difference (LSD) was used to separate treatment means whenever the *F* value was significant. The association Cochran–Mantel–Haenszel test was used to determine the relationship between feed supplementation and the incidence of intestinal lesions using the FREQ procedures. The difference between treatments was considered significant at a *P* < 0.05.

## Results

### Birds' Performance

Table 1 presents the composition of the all-vegetarian feed used in this study. Analyses of dry matter (DM), TP, amino acids, fatty acids, vitamins, and some of the most common minerals of the feed were performed at the Laboratory of Agro-Environmental Analysis (Table 2). Effects of the control and its supplementation with BACI, 1 and 2% organic cranberry (CP1, CP2) or wild blueberry (BP1, BP2) pomaces, as well as ethanolic extracts of cranberry (COH150, COH300) or blueberry (BOH150, BOH300) pomaces on BW, FI, FE, and mortality are illustrated in Table 3. The performance data obtained from this study showed improved phase dependent treatment effects on the BW and FE (Table 3).

**Table 1:** Composition of the feeds used in the present study.

**Ingredient (% of inclusion in diet)**	**Starter (days 0–10)**	**Grower (days 10–20)**	**Finisher (days 20–30)**
Corn	58.04	61.56	63.34
Soya	10.00	15.00	20.00
Soybean cake granule	13.10	7.40	3.90
Dresses distillery	5.00	6.00	6.00
Corn gluten	4.60	3.40	1.50
Canola oilcake	4.80	2.00	–
Limestone	1.53	1.50	1.42
Monocalcium phosphate	1.25	1.19	1.08
Soybean oil	–	0.40	1.20
Lysine sulfate 70%	0.43	0.39	0.34
Sodium bicarbonate	0.36	0.27	0.28
Salt	0.25	0.24	0.24
Luzern concentrate	0.20	0.20	0.20
Methionine	0.18	0.18	0.23
Myco-curb liquid	0.10	0.10	0.10
Choline liquid 75%	0.06	0.06	0.05
Hy D premix (Vitamin D3)	0.03	0.04	0.04
Threonine 98%	–	0.01	0.04
OptiPhos 1,000ct 250 ftu (0.12%)	0.03	0.03	0.03
Vitamin E 100,000 IU	0.05	0.03	0.02

**Table 2:** Analyzed nutrient profile of the feeds used in the present study.

**Nutrient**	**Starter (days 0–10)**	**Grower (days 10–20)**	**Finisher (days 20–30)**
**Calculated nutrient**			
Granulometry (μ)	1,362.54	1,324.14	1,299.26
Gross protein (%)	21.00	19.28	18.07
AMEn poultry (kcal/kg)	2,989.03	3,086.25	3,177.03
Phosphorus available (%)	0.50	0.48	0.45
Total chloride (%)	0.21	0.21	0.21
Total sodium (%)	0.22	0.19	0.19
Choline added (mg/kg)	396.99	396.99	351.43
Vit.A added (IU/kg)	11,000.00	10,100.00	10,100.00
Vit.D added (IU/kg)	4,988.24	4,984.32	4,984.32
Vit.E added (IU/kg)	80.00	60.00	50.00
Arginine (%)	1.26	1.17	1.14
Lysine (%)	1.23	1.13	1.08
Meth and Cys (%)	0.90	0.84	0.83
Methionine (%)	0.53	0.50	0.50
Threonine (%)	0.79	0.74	0.72
Tryptophane (%)	0.24	0.22	0.22
Arg Dig V Vol (%)	1.16	1.07	1.05
Lys Dig V Vol (%)	1.08	0.99	0.95
M and C Dig V Vol (%)	0.80	0.75	0.74
Met Dig V Vol (%)	0.49	0.47	0.48
Thr Dig V Vol (%)	0.67	0.62	0.61
Try Dig V Vol (%)	0.21	0.19	0.19
ValDVV/LDV (ratio)	0.76	0.77	0.76
Calcium phytase (%)	1.00	0.96	0.90
**Estimated nutrient**			
Dry matter (%)	89.80	89.70	90.10
Total protein (%)	23.06	20.44	18.69
C (%)	41.00	41.10	41.40
N (%)	3.69	3.27	2.99
C/N ratio	11.10	12.60	13.90
P (mg/kg)	8,015.00	7,542.00	7,307.00
K (mg/kg)	9,519.00	8,470.00	8,631.00
Ca (mg/kg)	9,041.00	9,954.00	11,053.00
Mg (mg/kg)	2,127.00	1,842.00	1,845.00
Na (mg/kg)	1,898.00	1,957.00	2,195.00

**Table 3:** Effects of cranberry and blueberry pomaces and their extracts on broiler growth performance and mortality^*^.

**Parameters**	**Control**	**Bacitracin**	**CP1**	**CP2**	**COH150**	**COH300**	**BP1**	**BP2**	**BOH150**	**BOH300**	**SEM**	***P*-value**
**Bodyweight, g/bird**												
Day 0	42.84	42.89	42.86	43.01	43.44	42.6	43.01	42.79	43.11	43.44	0.83	0.6
Day 0–10	241.41^C^	242.73^B, C^	242.86^B, C^	247.49^B^	248.76^B^	250.57^A^	241.01^C^	240.21^C^	245.93^B, C^	238.86^D^	0.001	0.01
Day 10–20	856.44^B, C^	866.93^B^	826.39^C^	768.84^D^	845.36^C^	858.07^B, C^	853.24^B, C^	856.01^B, C^	857.37^B, C^	869.64^A^	2.22	0.03
Day 20–30	1799.17	1817.47	1762.2	1762.2	1816.23	1815.47	1761.67	1797.09	1785.13	1810.24	1.37	0.22
**Feed intake, g/bird**												
Day 0–10	28.37	27.77	28.31	29.71	28.09	28.4	28.09	29.13	28.79	28.03	1.77	0.09
Day 10–20	81.53	77.01	81.19	82.4	72.25	74.51	82.61	86.27	82.53	82.9	0.78	0.64
Day 20–30	149.93	150.03	149.93	150.43	150.21	152.1	149.63	157.43	149.5	147.77	1.58	0.14
Day 0–30	928.57	900	928.57	928.57	900	885.71	957.14	985.14	928.57	942.85	0.002	0.25
**Feed efficiency, FE**												
Day 0–10	1.29^A, B^	1.26^B, C^	1.28^A, B^	1.32^A^	1.24^B, C^	1.23^B, C^	1.29^A, B^	1.34^A^	1.28^A, B^	1.3^A^	3.38	0.001
Day 10–20	1.46	1.36	1.54	2.07	1.34	1.35	1.49	1.57	1.49	1.45	1.37	0.22
Day 20–30	1.58	1.59	1.61	1.58	1.54	1.58	1.64	1.66	1.62	1.58	1.06	0.41
Day 0–30	1.5^B^	1.47^C^	1.55^A, B^	1.59^A^	1.44^C^	1.46^C^	1.55^AB^	1.58^A^	1.53^B^	1.5^B^	2.68	0.01
Mortality (%)	1.43	3.57	2.14	4.29	4.64	3.21	2.5	3.57	2.86	2.86	1.1	0.38

* *Berry products and bacitracin were administrated via feed from 0 to 30 days. Data represent means ± SEM of seven replicates/treatment (n = 7 pens of at least 40 chickens/pen) arranged in a completely randomized block design. P-value was obtained by ANOVA. Different superscripted capital letters within a row indicate significant differences at P < 0.05*.

During the starter phase, the highest BW (*P* < 0.05) was observed in birds treated with COH300, COH150, and CP2. A similar BW was found in birds fed BOH150, CP1, and BACI. No significant difference was found between the control birds and those fed BP1 and BP2, while the lowest BW was recorded in the BOH300 treatment. During the grower phase, the highest (*P* < 0.05) BWs were obtained with the BOH300 and BACI treatment, whereas no significant differences were observed between COH300, BOH150, control, BP2, and BP1. The lowest BW values were recorded in the COH150-, CP1-, and CP2-treated birds (*P* < 0.05). However, no statistical difference was found among the 10 treatment groups for the cumulative (overall) final BW.

In the starter phase, COH300, COH150, and BACI feed treatments induced the lowest (improved) FE, while BP2, CP2, and BOH300 induced the highest (poorer) FEs (*P* < 0.05). No significant effect was observed between the control birds and those fed BP1, CP1, and BOH150. At day 30, COH150-, COH300-, and BACI-fed birds induced the best cumulative FE values among all feed treatments (*P* < 0.05). No significant effects of pomaces or their ethanolic extracts in feed were observed on the cumulative (days 0–30) FI or the mortality rate compared to control and BACI.

### General and Intestinal Health

Gross examination revealed that the general heath was good; bone, cartilage, as well as muscle quality was adequate. At day 21 necropsy, few lesions on the internal organs, cases of retained yolks, and very slight airsacculitis were observed, with no evidence of active infection. Only three birds (4.3%) with airsacculitis were found in each of the BACI and BOH150 treatments. The bursae of Fabricius were in good condition with a satisfactory size, indicating a functioning immune system and absence of a health challenge.

At day 21, liquid and mucous intestinal content was observed in the majority of birds necropsied, which could be partially explained by the effect of the all-vegetarian diet used. In general, subclinical (minor low lesion scores) NEs were observed, as shown in Table 4. The lowest (*P* < 0.05) prevalence of NE score of 1 (occasional lesions consisting of small areas of erosion, necrosis, or hemorrhage) was observed in birds treated with CP1 (21.4%) and BP1 (21.4%) compared to the BACI (42.8%) and control (42.8%) treatments. Only one bird with an NE lesion score of 2 (minor gross lesions consisting of occasional small areas of hemorrhage or necrosis at one to two lesions per 5 cm of the small intestine) was observed in the control group. In almost all necropsied birds independently of treatment, a

**Table 4:** Prevalence of birds presenting lesion scores: coccidiosis due to *Eimeria* spp. and necrotic enteritis (NE) caused by Clostridium perfringens^*^.

**Treatments**	***Eimeria acervulina***		***Eimeria maxima***		***Eimeria tenella***		**NE**	
	**1**	**2**	**1**	**2**	**1**	**2**	**1**	**2**
Control	28.57	28.57	28.57	0	42.86	7.14	42.86	7.14
Bacitracin	42.86	21.43	21.43	0	35.71	14.29	42.86	0
CP1	42.86	21.43	7.14	0	28.57	7.14	21.43	0
CP2	42.86	28.57	14.29	0	7.14	14.29	78.57	0
COH150	50	14.29	21.43	0	0	21.43	78.57	0
COH300	57.14	7.14	14.29	0	7.14	7.14	28.57	0
BP1	42.86	28.57	21.43	0	35.71	7.14	21.43	0
BP2	50	7.14	0	7.14	35.71	7.14	50	0
BOH150	21.43	21.43	7.14	7.14	35.71	0	57.14	0
BOH300	42.86	28.57	7.14	0	28.57	7.14	50	0
*P-*value	0.88		0.94		0.58		0.01	

* *Two birds per pen (14/treatment, 140 birds total) were sacrificed on days 21–22 for necropsy*.

*E. acervulina causing white plaques in the duodenum; the scores were scored on a scale of 0–4: “0”—normal, “1”—a maximum of five lesions per cm^2^ mainly in the duodenum, “2”—several lesions in the duodenum and/or jejunum, but not coalescent*.

*E. maxima induces bleeding in the middle of the small intestines, scored from 0 to 4 as follows: “0”—normal, “1”—few petechiae on the serosal surface around Meckel's diverticulum, or in other areas of the intestine, “2”—several petechiae on the serosal surface, small petechiae on the mucosal side, watery contents, orange intestinal mucus*.

*E. tenella causing severe inflammation of ceca includes intestinal score from 0 to 4: “0”—normal, “1”—few petechiae on the cecal serosal and mucosal surfaces or little blood in the ceca and thick cecal contents, “2”—petechiae on the cecal serosal and mucosal surfaces or thick cecal wall or contents containing blood or fibrin and presence of grooves*.

*C. perfringens was scored on a scale of 0 to 3: “0”—no gross lesions, “1”—occasional lesions consisting of small areas of erosion, necrosis, or hemorrhage, “2”—minor gross lesions consisting of occasional small areas of hemorrhage or necrosis at one to two lesions per 5 cm^2^ throughout the small intestine*.

duodenal congestion was notable, and in some birds, a lesion score of 1 or 2 due to *E. acervulina* was observed. The lowest but not significant (*P* = 0.94) prevalence of intestinal lesion scores of 1 (few petechiae on the serosal surface around Meckel's diverticulum or in other areas of the intestine) by *E. maxima* was observed in birds treated with CP1, BOH150, and BOH300 (7.1% for each treatment) compared to the control- (28.6%) and BACI- (21.4%) treated birds. Among all treatments, the prevalence of *E. tenella* was lower in the CP2 and COH300 groups (7.1% for each treatment) than that in the control (42.8%). At day 28 of age, no necropsy was conducted, due to the general good health status of birds.

### Blood Serum Metabolites

Nineteen blood serum metabolite levels were measured in birds at days 21 and 28 of age, which showed significant treatment effects for several biomarkers (Table 5). On day 21, BOH300 significantly reduced (*P* < 0.05) the serum enzymes ALT and LIP levels. The highest level (*P* = 0.08) of ALP was observed in birds treated with BACI and COH150. Compared to control birds, BOH300-treated birds showed 85 and 50% lower ALT and LIP content, respectively. Except the BOH300 treatment, the ALT levels decreased with age in all treatment groups, including the control. At day 21, significant treatment effects were observed for Ca, P, and Mg concentrations, with the lowest level of these three minerals (Ca = 1.58 mmol/L, P = 1.89 mmol/L, Mg = 0.93 mmol/L) being observed in CP2-fed birds (*P* < 0.05). Both levels of TRIG and NEFA were significantly decreased (~20 and 16%, respectively) in all cranberry by-product–treated birds compared to control, with the lowest level of AGR being observed in birds treated with BACI, COH300, and COH150 (*P* < 0.05). Similar to day 21, ALT level was significantly low (*P* < 0.001) at day 28 in blueberry product–treated birds. Among minerals, only Mg was influenced (*P* < 0.05) by all cranberry by-products compared to control and the blueberry by-product treatments at day 28. Levels of TRIG and ALB (protein) were significantly low (*P* < 0.05) in birds treated with cranberry pomace compared to the other treatments in 28-day-old birds. Calculated AST:ALT ratio values were high on both days 21 and 28, with the highest values observed in blueberry pomaces and their ethanolic extract–fed chickens.

**Table 5:** Blood serum metabolites of broiler chickens fed with organic cranberry (CP), wild blueberry (BP) pomace (1–2%), and their respective ethanolic extracts (COH150, COH300, BOH150, and BOH300 ppm) at days 21–28^*^.

**Age (day)**	**Categories**	**Metabolites**	**Treatments**											
			**Control**	**Bacitracin**	**CP1**	**CP2**	**COH150**	**COH300**	**BP1**	**BP2**	**BOH150**	**BOH300**	**SEM**	***P*-value**
21	Serum enzymes (U/L)	Alanine aminotransferase (ALT)	8.67	8.50	9.14	7.50	7.86	7.86	1.57	1.57	1.86	1.29	0.57	<0.001
		Aspartate aminotransferase (AST)	322.17	288.17	239	210.17	298.29	239.71	234.86	314.86	248.86	247.57	33.03	0.255
		Alkaline phosphatase (ALP)	5,884	11,148.5	9,950	10,099.67	13,527.29	7,538.14	10,664.29	10,586.43	10,625.71	10,407.14	1,503.70	0.083
		Amylase (AMY)	917.33	1,332.83	1,091.43	691.17	1,075.14	775.57	1,034	850.71	883.86	779.43	176.38	0.362
		Lipase (LIP)	17.33	21.00	16.71	15.67	17.00	14.29	11.86	9.00	10.43	8.71	1.99	0.000
		Gamma-glutamyltransferase (GGT)	9.00	11.17	8.86	7.83	7.43	5.14	13.57	11.14	9.00	8.57	1.88	0.143
	Mineral (mmol/L)	Calcium (Ca)	1.92	1.94	2.06	1.58	2.05	1.95	1.83	2.12	1.95	2.08	0.11	0.057
		Magnesium (Mg)	1.07	1.03	1.07	0.93	1.16	1.03	1.00	1.11	1.11	1.07	0.06	0.047
		Phosphorous (P)	2.27	2.12	2.14	1.89	2.47	2.20	2.08	2.50	2.47	2.41	0.14	0.057
		Iron (Fe)	15.5	18.17	16.14	16.17	16.86	17.71	14.00	16.14	17.00	15.71	1.25	0.532
	Carbohydrate (mmol/L)	Glucose	14.68	14.48	13.97	13.12	14.3	12.93	14.00	13.31	14.61	14.83	0.74	0.567
	Lipid (mmol/L)	Cholesterol (CHO)	2.90	3.02	2.94	2.59	2.95	2.73	2.99	2.92	2.99	2.99	0.20	0.914
		High-density lipoprotein cholesterol (HDLC)	2.22	2.24	2.27	1.99	2.24	2.26	2.19	2.18	2.25	2.23	0.14	0.971
		Triglyceride (TRIG)	0.88	0.98	0.70	0.70	0.77	0.73	0.90	0.96	1.11	1.19	0.09	0.003
		Non-esterified fatty acids (NEFA)	0.62	0.70	0.53	0.53	0.71	0.52	1.04	0.93	1.20	1.08	0.09	<0.001
	Protein (g/L)	Total protein	22.83	25.17	23.43	22.00	23.29	21.71	22.71	23.57	24.00	23.57	1.35	0.860
		Albumin (ALB)	11.17	10.83	11.00	10.83	10.71	9.57	12.00	11.57	12.29	11.71	0.67	0.224
		Globulin (GLO)	11.67	14.33	12.43	11.17	12.57	12.14	10.71	12.00	11.71	11.86	0.80	0.215
		ALB–GLO Ratio (AGR)	0.97	0.77	0.90	0.96	0.85	0.79	1.13	0.99	1.05	1.01	0.048	<0.001
28	Serum enzymes (U/L)	ALT	5.60	6.40	6.20	3.80	4.60	3.80	1.40	1.00	1.60	1.60	0.74	<0.0001
		AST	215.75	236.40	273.80	205.2	220.80	229.00	229.40	232.40	210.80	274.00	26.25	0.592
		ALP	5,668.80	5,692.40	6,858.00	5,540.80	6,269.80	8,608.00	5,174.80	6,793.60	7,258.40	7,507.80	1,719.79	0.934
		AMY	1,131.80	767.00	849.40	856.40	643.20	473.20	931.00	801.80	761.60	921.40	267.46	0.908
		LIP	17.20	15.60	13.60	13.40	11.60	11.60	11.40	7.00	7.60	10.60	2,512.00	0.145
		GGT	8.20	10.40	9.80	12.20	9.60	14.00	13.40	14.60	13.40	10.80	1.99	0.337
	Mineral (mmol/L)	Ca	2.31	1.98	1.93	1.87	1.94	1.90	1.95	1.87	1.87	2.05	0.14	0.568
		Mg	1.04	0.96	0.86	0.82	0.88	0.84	0.94	0.92	1.00	0.94	0.05	0.044
		P	2.45	2.16	1.88	1.97	2.10	1.94	2.11	2.01	2.15	2.31	0.16	0.384
		Fe	17.4	17.2	15.2	13.8	16.80	15.00	18.2	17.8	15.40	17.20	1.38	0.389
	Carbohydrate (mmol/L)	Glucose	14.44	13.96	13.24	12.80	15.02	14.00	13.50	14.52	11.46	14.28	1.05	0.486
	Lipid (mmol/L)	CHO	3.25	3.1	2.76	2.36	2.92	2.89	3.17	2.97	2.80	3.21	0.20	0.109
		HDLC	2.14	2.25	2.06	1.77	2.18	2.14	2.40	2.33	1.93	2.39	0.18	0.299
		TRIG	1.20	0.92	0.80	0.84	0.82	0.96	1.12	0.92	1.02	1.08	0.086	0.024
		NEFA	1.23	0.87	0.70	0.81	0.83	0.89	1.11	1.00	1.19	1.25	0.156	0.148
	Protein (g/L)	Total protein	26.60	24.00	22.80	21.60	23.00	22.60	26.60	24.60	25.80	25.20	1.77	0.460
		ALB	14.40	11.60	10.40	10.20	11.40	11.00	12.80	12.60	12.80	13.40	0.73	0.003
		GLO	12.20	12.40	12.40	11.40	11.60	11.60	13.80	12.00	13.00	11.80	1.21	0.945
		AGR	1.35	0.94	0.87	0.95	0.99	0.97	0.93	1.05	0.99	1.14	0.19	0.242

* *Berry products were administrated via feed from 0 to 30 days. Data represent means ± SEM of seven replicates/treatment (n = 7 pens of at least 40 chickens/pen) arranged in a completely randomized block design. P-value obtained by ANOVA*.

### Plasma Metabolomics

**Figure 1:**
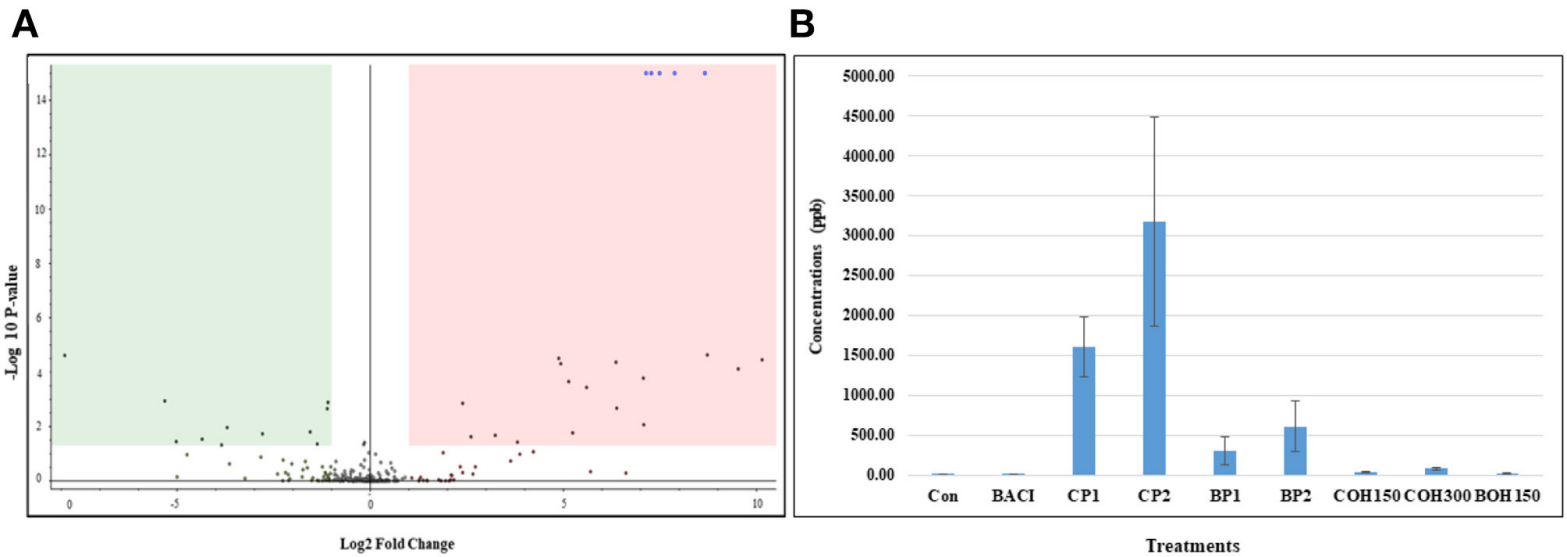
**(A)** Volcano plot generated by differential analysis and showing a representative metabolomics profile of blood plasma of chickens treated with control and cranberry 2%. Volcano plot of fold changes (x-axis) and their associated log_10_ transformed *P*-values (y-axis) for phenolic compounds analyzed by LC-MS. The green and pink area indicated downregulation and upregulation, respectively. The blue dots in the upregulated area were identified as the quinic acid. **(B)** Quinic acid level in blood plasma of chicken fed with or without berry products. Data represent means ± SEM of seven replicates/treatment (two birds/pen = 14 birds/treatment).

The effect of feed supplementation with berry pomaces and their ethanolic extracts was evaluated on 140 chicken (two birds/pen) blood plasma samples (seven pens/treatment) at day 21 of age. Significant differences (*P* < 0.05) were noticed between treatments. Compared to control birds, all berry by-product–fed birds showed downregulation (green area) or upregulation (pink area) of the concentration of several metabolites as shown on the Volcano plot by differential analysis (Figure 1A). The blue dots in the upregulated area were identified and confirmed as quinic acid (QA) 1,3,4,5-tetrahydroxy-1-cyclohexanecarboxylic acid, concentrations of which were clearly higher in CP1 and CP2 compared to other treatments (Figure 1B).

### Culture Dependent Bacteriology

Chicken ceca from all treatment groups were used for bacterial enumeration on selective media. In the ceca of 21-day-old birds, significantly lower populations of *C. perfringens* and *Escherichia coli* and higher counts of *Lactobacillus* spp. were observed in birds fed diets supplemented with berry by-products than control-fed birds except for COH300 feed groups (Table 6). The effect of CP2 appeared to be comparable to that of BACI at 55 ppm, especially in lowering *C. perfringens* and *E. coli* counts in the ceca. CP2 and COH300 significantly decreased the population of *E. coli*, whereas CP1, CP2, and COH150 significantly decreased *C. perfringens* counts, Higher counts of *C. perfringens* and *E. coli* were observed in blueberry pomaces and their by-products compared to both control (basal diet and bacitracin) and cranberry by-product treatment groups. The population size of *Lactobacillus* spp. was significantly higher in all birds fed blueberry products and CP2 (*P* < 0.05) than those of control and BACI treatment, but lower in the COH300-treated birds.

**Table 6:** Log_10_ bacterial numbers per gram of cecum samples from broiler chickens under berry pomaces and their ethanolic extracts.

**Bacteria**	**Control**	**Bacitracin**	**CP1**	**CP2**	**COH150**	**COH300**	**BP1**	**BP2**	**BOH150**	**BOH300**	**SEM**	***P*-value**
*Escherichia coli*	3.89	1.67	3.60	1.25	3.06	1.03	4.04	3.92	3.96	3.98	0.528	<0.0001
*Lactobacillus*	6.62	5.99	6.98	7.27	6.16	1.03	7.45	7.35	7.23	7.09	0.528	<0.0001
*C. perfringens*	1.57	0.39	0.46	0.00	0.33	2.09	2.90	3.08	3.00	2.60	0.478	<0.0001

*Standard error of means of seven replicates/treatment (n = 7 pens of 2 chickens/pen) arranged in a completely randomized block design*.

### 16S rRNA Gene Sequencing

After quality filtering, a total of 4,576,860 and 4,359,267 sequence reads were obtained from the cecal and cloacal samples, respectively, with the total reads per sample ranging from 29,449 to 110,189 in ceca and 30,460 to 98,801 in cloacae. On average, 62,381 sequence reads per sample were generated, averaging 356 and 252 OTUs in the ceca and cloacae, respectively (Table 7). The mean of Good's coverage (an alpha diversity index) for all samples was high (>98%), indicating that the majority of the microbial phylotypes in the ceca and cloacal samples were covered.

**Table 7:** Summary statistics of sequences analyzed including the number of average sequences after filtering but before operational taxonomy unit (OTU) picking, average reads after OTU picking, average OTU numbers, and microbial diversity covered.

**Site**	**Treatment**	**Average reads/sample**	**Average OTUs**	**(% Good's coverages)**
Cecum	Control	50,624.14	366.29	0.999
	Bacitracin	54,685.43	346.00	0.999
	CP1	51,922.57	364.86	0.999
	CP2	62,884.57	371.14	0.998
	COH150	79,487.57	369.71	0.999
	COH300	77,798.14	368.43	0.998
	BP1	77,517.14	365.14	0.999
	BP2	52,605.86	285.86	0.999
	BBOH150	62,950.29	378.71	0.999
	BBOH300	53,335.43	345.14	0.998
Cloaca	Control	55,710.57	259.00	0.998
	Bacitracin	72,261.14	303.43	0.998
	CP1	53,229.29	189.86	0.999
	CP2	54,064.86	241.43	0.998
	COH150	69,677.43	268.29	0.998
	COH300	71,845.29	254.14	0.998
	BP1	68,619.86	246.29	0.998
	BP2	49,943.43	254.86	0.998
	BBOH150	47,803.86	221.86	0.998
	BBOH300	57,764.86	290.29	0.998

*For each of seven feed treatments, the sequencing reads were merged, and OTUs were clustered at >97% similarity using Quantitative Insights Into Microbial Ecology (QIIME)*.

### Species Richness and Diversity in Ceca and Cloacae

Alpha diversity indices of both cecal and cloacal data showed similar values for all 10 treatments. An increase in species richness and evenness was observed in the cecum, compared to cloaca, as indicated by both Chao1 and Shannon metrics. At the phylum level, the higher relative abundances of unassigned sequences, *Actinobacteria, Bacteroidetes, Proteobacteria*, and *Tenericutes* were observed in ceca compared to the cloacal microbial community (*P* < 0.05). Interaction between sample sources (cecum or cloaca) and treatments was only noticed at the phylum level for the unassigned and *Actinobacteria*. The PCoA plot using Permanova for Unifrac weighted β-diversity demonstrated no clustering (*P* > 0.05) either in the cecum or in the cloaca for any of the treatment groups at day 21 of age.

### Cecal Microbial Population

**Figure 2:**
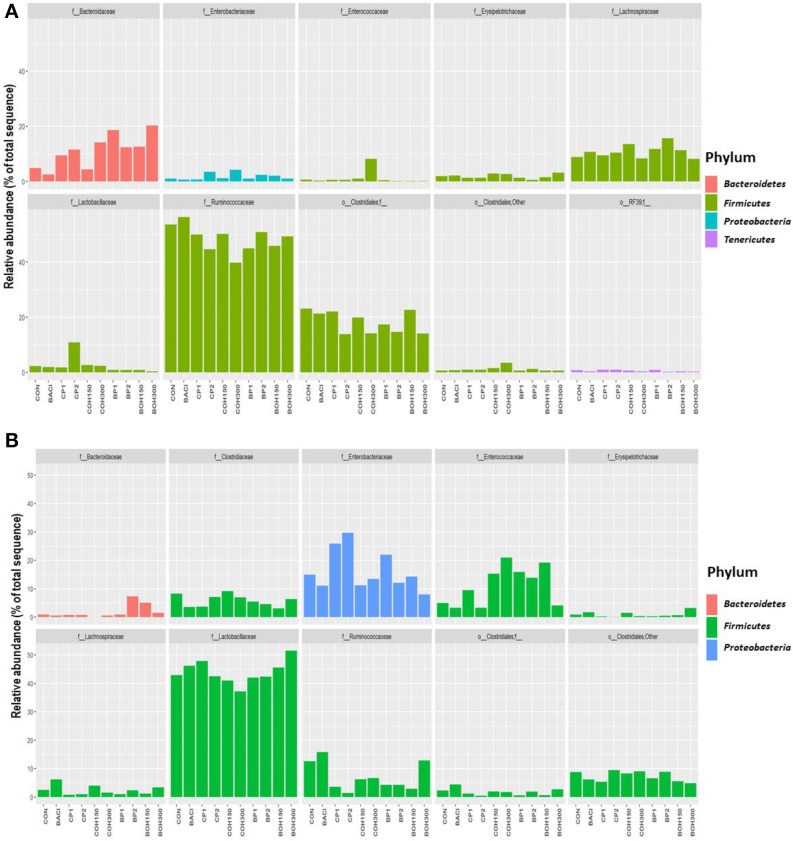
**(A)** Relative abundances of the top 10 families averaged over all samples for the feed supplement groups for the ceca. **(B)** Relative abundances of the top 10 families averaged over all samples for the feed supplement groups for the cloaca.

**Figure 3:**
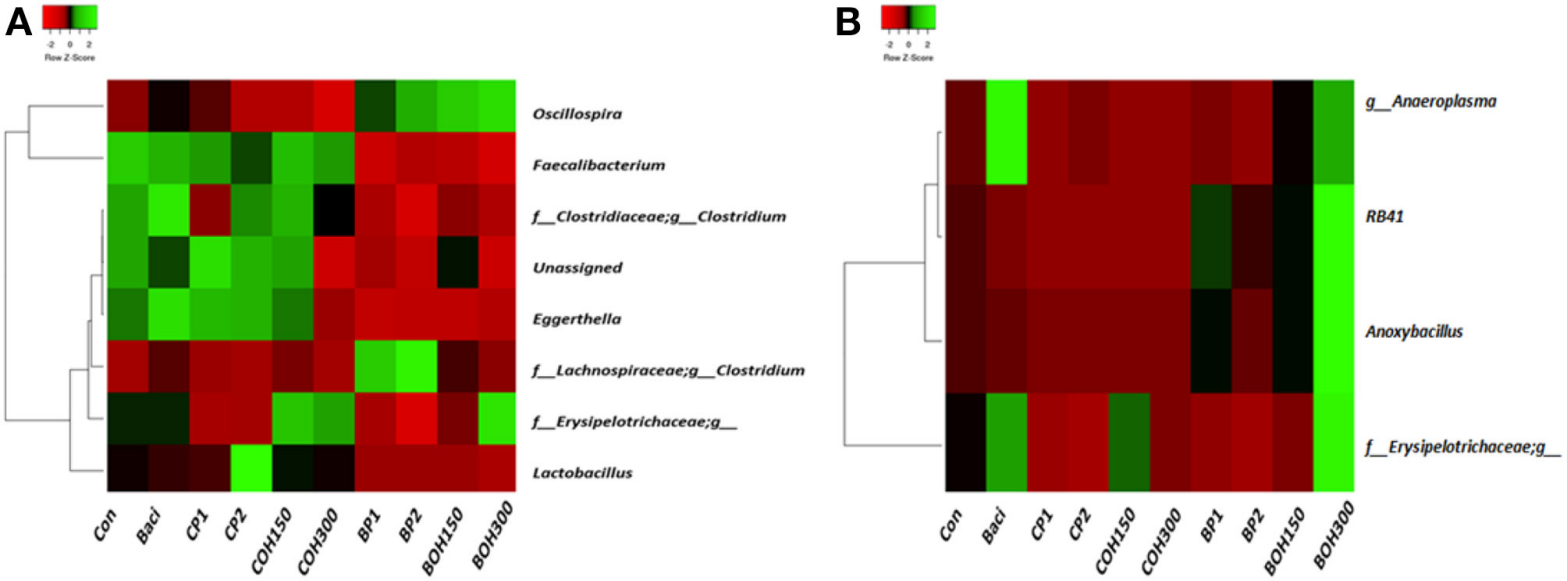
Heatmap showing bacterial genera whose relative abundances were significantly (*P* < 0.05) changed by studied dietary treatments in **(A)** ceca and **(B)** cloacae.

The phyla with the highest relative abundances (≥1%) in 21-day-old broilers were *Firmicutes* (85.4%) and *Bacteroidetes* (11.1%), while *Proteobacteria* and *Tenericutes* were present at 1.8 and 1.1%, respectively. Significant effects (*P* < 0.05) were observed with BACI and CP2 treatments on the relative abundance of *Actinobacteria* (Table 8A). *Tenericutes* tended (*P* = 0.08) to be influenced by CP1 feeding (Table 8A). Across all treatments, the predominant bacterial families (≥1%) in ceca of 21-day-old broilers were *Ruminococcaceae* (48.6%), *Clostridiales* (18.3%), *Bacteroidaceae* (11.1%), *Lachnospiraceae* (10.9%), *Lactobacillaceae* (2.5%), and others (Figure 2A). Significant cranberry pomace effects (*P* < 0.05) were observed on the abundance of bacterial phyla *Eggerthella* (*Coriobacteriaceae_f), Lactobacillus (Lactobacillaceae_f), Faecalibacterium (Ruminococcaceae_f)*, and *f__(Mogibacteriaceae)*. *Lachnospira* and *Coprococcus* (from the *Lachnospiraceae_*family) were affected by the blueberry pomace treatments, while *Oscillospira (Ruminococcaceae_f) and Erysipelotrichaceae_f* were affected by the BOH300 treatments (Figure 3A). *Lactobacillaceae* were significantly higher in the ceca of birds fed CP2 compared to BACI and the control-treated groups (Figures 2A, 3A). The highest OTUs (*P* < 0.05) classified as *Lactobacillus agilis* (4.3%) and other unidentified *Lactobacillus* spp. (6.6%) known to include some isolates with probiotic activity were found largely in CP2-treated birds. BACI treatment affected the abundances of both *Clostridium* and *Eggerthella* (Figure 3A). *Enterococcus* spp., *L. agilis*, and *Blautia producta* were some unique bacterial species found only in the cranberry by-product treatments compared to BACI feed treatment. Accordingly, dietary COH300 was found to increase the abundance (8.0%) of *Enterococcus* compared to other treatments.

**Table 8:** Relative abundance of bacterial phyla treated with different feed supplements at **(A)** ceca and **(B)** cloacae of broiler chickens at 21 days of age^1^.

**Phylum**	**Control**	**Bacitracin**	**CP1**	**CP2**	**COH150**	**COH300**	**BP1**	**BP2**	**BOH150**	**BOH300**	***P*-value**
**(A) Cecum (%)**											
Unassigned	0.24^A, B, C^	0.20^A, B, C, D^	0.34^A^	0.26^A, B^	0.23^C, D, E^	0.11^D, E^	0.13^A, B, C^	0.08^E^	0.19^B, C, D, E^	0.06^E^	0.001
*Actinobacteria*	0.11^A, B, C^	0.19^A^	0.15^A, B^	0.14^A^	0.11^c^	0.06^C^	0.03^A, B, C^	0.03^B, C^	0.03^C^	0.04^c^	0.003
*Bacteroidetes*	4.93	2.6	9.46	11.52	4.36	14.18	18.67	12.36	12.62	20.27	0.327
*Cyanobacteria*	0.2	1.05	0.22	0.09	0.11	0.24	0.16	0.18	0.05	0.16	0.46
*Firmicutes*	92.07	94.54	87.07	83.45	92.75	80.11	78.74	84.72	83.98	76.78	0.286
*Proteobacteria*	1.11	0.73	0.81	3.57	1.16	4.4	1.12	2.43	2.09	1.1	0.35
*Tenericutes*	1.33	0.69	1.95	0.97	1.27	0.9	1.15	0.2	1.04	1.59	0.087
**(B) Feces (%)**											
Unassigned	0.36	0.31	0.25	0.25	0.19	0.42	0.34	0.17	0.25	0.13	0.07
*Acidobacteria*	0.01^B^	0.00^B^	0.00^B^	0.00^B^	0.00^B^	0.00^B^	0.03^B^	0.01^B^	0.02^B^	0.12^A^	0
*Bacteroidetes*	0.93	0.61	0.78	0.99	0.15	0.56	0.9	7.36	5.16	1.5	0.3
*Cyanobacteria*	0.23	0.11	0.21	0.78	0.38	0.41	0.21	1.06	0.51	0.28	0.32
*Firmicutes*	83.48	87.81	72.85	65.47	88.01	85.05	76.48	79.09	79.65	89.82	0.36
*Proteobacteria*	14.92	10.98	25.9	32.49	11.21	13.54	22.01	12.25	14.37	7.95	0.19
*Tenericutes*	0.06	0.16	0.02	0.02	0.06	0.01	0.03	0.04	0.03	0.19	0.09

1 *n= 7 pens/treatment, 2 birds/pen: 14 birds/treatment*.

A−E *Means with different superscripts within a row differ significantly (P < 0.05)*.

### Cloacal Microbial Population

The most abundant phyla (≥1%) in 21-day-old broiler cloacal samples were *Firmicutes* (80.8%), followed by *Proteobacteria* (16.6%) and *Bacteroideteses* (1.9%), while other phyla were present at substantially lower levels (<1%). At the phylum level, a significant treatment effect (*P* < 0.005) was observed for BOH300, which increased the relative abundance of *Acidobacteria* compared to any other treatment (Table 8B). Similar to ceca, *Firmicutes* were the most abundant phyla, with the highest and lowest relative abundances being found with BOH300 (89.9%) and CP2 (65.4%), respectively (Table 8B). *Lactobacillales* (55.0%) and *Clostridiales* (24.5%) were the major orders found within *Firmicutes*, whose relative abundances slightly varied with different feed treatments (*P* < 0.05). Three *Clostridiales* families—*Clostridiaceae* (5.8%), *Lachnospiraceae* (2.4%), and *Ruminococcaceae* (7.1%)—predominated (Figure 2B), whereas *Lactobacillaceae* (43.9%) and *Enterococcaceae* (11.0%) were the most abundant orders in *Lactobacillales* group (Figure 2B). At the family level, BOH300 significantly (*P* < 0.05) affected the relative abundances of *RB41;f_, Bacillaceae*, and *Erysipelotrichaceae* (Figure 2B), while the population of *Anaeroplasmataceae* was influenced by the BACI treatment. The top genera belonging to the order *Lactobacillales* and *Clostridiales* were predominated by *Lactobacillus* (43.9%), *Enterobacteriaceae_* (15.9%), and *Enterococcus* (10.7%). A significant treatment effect was mostly observed with the blueberry by-product treatment; especially, BOH300 significantly (*P* < 0.05) increased the relative abundances of *Anoxybacillus kestanbolensis* and *Erysipelotrichaceae_f* (Figure 3B). *Acinetobacter, Pseudomonas, Comamonas*, and *Stenotrophomonas* were some of the unique bacterial groups found only in cloaca samples of CP2-fed birds compared to other treatment groups.

### Correlation Between Cecal Taxa, Performance, and Blood Metabolites

**Figure 4:**
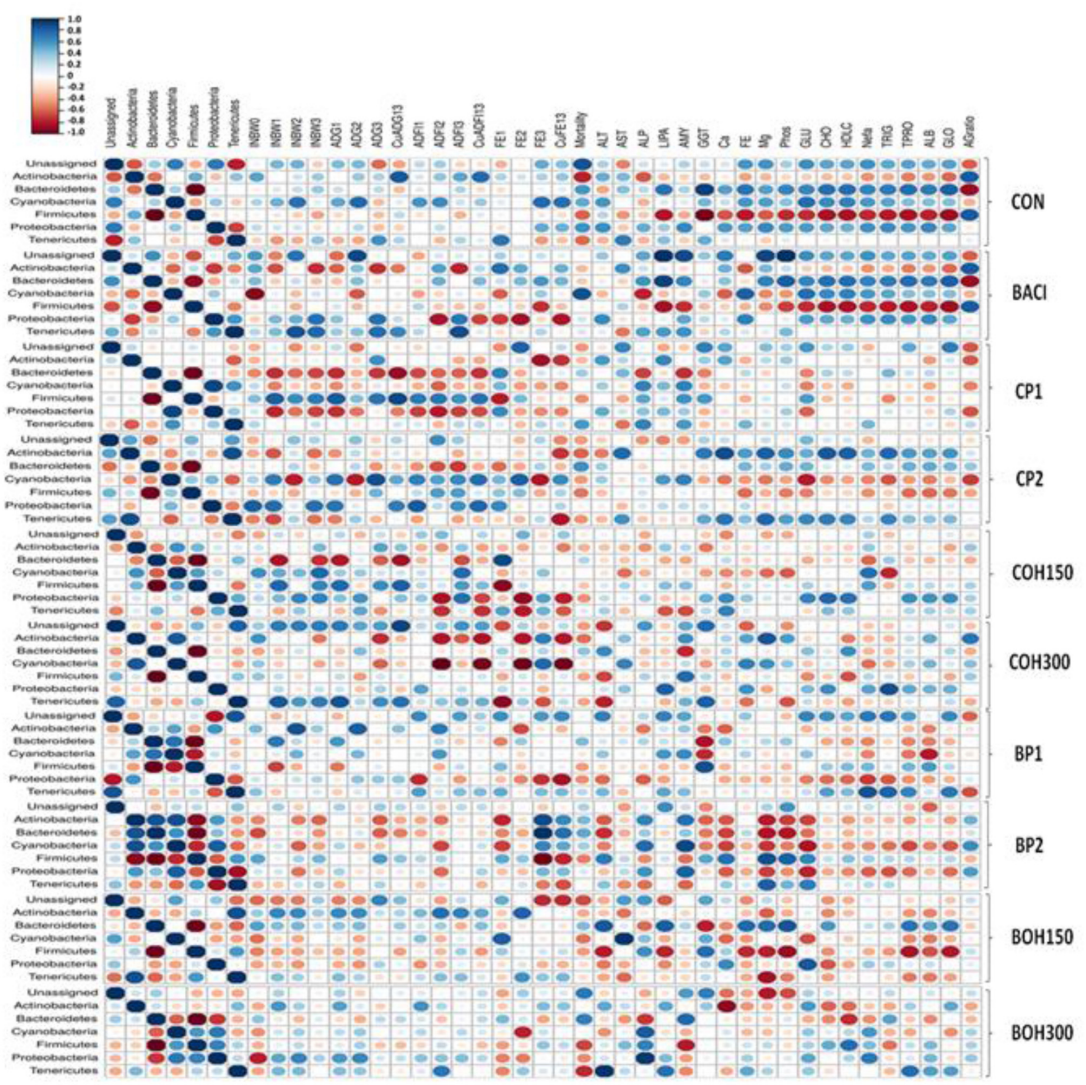
Spearman non-parametric rank correlations among bacterial phyla, performances [individual body weight (INBW), average daily gain (ADG), average daily feed intake (ADFI), feed efficiency (FE) in the starter, grower, and finisher phases as well as during the overall raising period (cumulative)]; mortality; and different blood metabolites: enzymes, minerals, glucose, lipids, and protein. The scale colors indicate whether the correlation is positive (closer to +1, blue circles) or negative (closer to −1, red circles) between the taxa (phylum), performances and the blood metabolites. All correlations presented were statistically significant (*P* < 0.05). Large and small circles indicate strong and weak correlations, respectively.

Significant correlation was observed between cecal bacterial phyla, several performance parameters, and blood metabolites (Figure 4). As expected, a consistent negative correlation (*P* < 0.05) was observed between *Firmicutes* and *Bacteroidetes* regardless of treatments. Other bacterial phyla showed diverse significant correlations with each other (*P* < 0.05). For example, *Actinobacteria* were negatively correlated with *Proteobacteria, Tenericutes*, and *Cyanobacteria* in BACI, CP2, and COH300 treatment groups, respectively. However, in the BP2-treated group, significant positive correlations of *Actinobacteria* with *Bacteroidetes* and *Cyanobacteria* were observed. A significant negative correlation was observed between *Tenericutes* and *Proteobacteria* in both the BP2 and control treatments (*P* < 0.05).

Across all treatments, no consistent correlations were observed between bacterial phyla and performance. The cumulative (overall) FE (CuFE13) was positively correlated with *Actinobacteria* in control-, BACI-, and BP2-treated birds, which was not observed with the cranberry pomaces and its ethanolic extracts. A positive correlation was found between *Tenericutes*, initial BW (INBW2), and average daily FI (ADFI2) during the grower period (*P* < 0.05) in BACI, COH300 and BOH300 treatments; however, both berry pomace treatments resulted in a negative correlation between *Tenericutes* and cumulative FE (CuFE13). Interestingly, *Actinobacteria* was negatively correlated with mortality in birds fed control and cranberry by-products (*P* < 0.05), suggesting that increasing *Actinobacteria* could be beneficial by decreasing the mortality rate. On the other hand, *Bacteroidetes* and *Cyanobacteria* were positively correlated with the mortality rate in berry pomace and BACI treatments, respectively.

In control- and BACI-fed birds, the phylum *Firmicutes* was negatively correlated with blood metabolites such as serum enzymes, fats, and TP. Accordingly, LIP, AMY, GGT, glucose, CHO, HDLC, TRIG, TPRO, and GLO were all negatively correlated (*P* < 0.05) with *Firmicutes*. Similar correlation patterns were also found with the CP2 treatments, particularly for the minerals and proteins. As stated above, at day 21 of age, a significant treatment effect was observed for enzymes ALT and LIP in chicken serum of birds treated with blueberry products. ALT was negatively correlated with *Actinobacteria* and *Bacteroidetes*, while *Firmicutes* showed a positive correlation with this ALT enzyme. Significant treatment effects for blood minerals (Ca and P), TRIG, and NEFA were found mainly in CP2-treated birds in blood. However, the correlation analysis showed positive correlations of *Actinobacteria* with either minerals or lipid and protein profiles. A positive correlation (*P* < 0.05) was observed between AGR and both *Actinobacteria* and *Firmicutes* in the BACI treatment.

### Expression of Innate and Adaptive Immune Genes in Spleen

Since cranberry product feeding appeared to induce the shifts in gut microbiota toward potential beneficial bacteria, only spleens from 21-day-old birds fed cranberry by-products were analyzed for expression of 84 immune genes. Out of the 84 analyzed genes, 13 genes were upregulated, but the *MX1* was downregulated in the spleen of birds fed BACI and cranberry products compared to control group. Among the 12 upregulated genes, cranberry product treatments upregulated expression of the Th2 type immune response genes including those encoding IL4, IL5, CSF2, and HMBS compared to control. BACI treatment induced expression of genes coding for CCR4, CRP, and IL13 belonging to Th2. Moreover, genes coding for JAK2 and TLR15 appeared to be upregulated in the cranberry-treated birds while downregulated in the BACI-treated birds compared to the control ones (*P* < 0.05), but the levels of their expression were less than two-fold (Table 9). Interestingly, the levels of expression of genes for JAK2 and TLR15 in cranberry product–fed birds appeared to be oppositely expressed compared to BACI-treated birds (Table 9). Overall, no linear dose response effect was observed with cranberry product feeding; however, CP1 seemed to have consistently higher gene expression levels.

**Table 9:** Differentially expressed genes of innate and adaptive immune response pathway from chicken spleen tissue in response to feed treatments compared to control.

**Gene symbol**	**Description**	**Fold change**				
		**BACI**	**CP1**	**CP2**	**COH150**	**COH300**
CCR4	Chemokine (C-C motif) receptor 4	2.07^*^	1.93	1.38	1.6	1.64
CCR5	Chemokine (C-C motif) receptor 5	−1.2	2.07	1.71	1.35	1.52
CCR6	Chemokine (C-C motif) receptor 6	1.03	2.04^*^	1.89	1.5	1.85^*^
CD14	CD14 molecule	2.43	2.20^*^	2.09	2.25	1.93
CRP	C-reactive protein, pentraxin-related	6.20^*^	1.97	1.84	2.47	2.2
CSF2	Granulocyte-macrophage colony-stimulating factor	4.07	3.74^*^	2.97^*^	2.79^*^	2.82^*^
IL4	Interleukin 4	2.98	2.31^*^	2.14^*^	2.1	2.45^*^
IL5	Interleukin 5	9.42	12.65^*^	10.84^*^	11.58^*^	12.06^*^
IL13	Interleukin 13	2.08^*^	1.36	1.12	1.18	1.13
HMBS	Hydroxymethylbilane synthase	4.89	2.93^*^	2.20^*^	1.91	2.06^*^
MX1	Myxovirus (influenza virus) resistance 1, interferon-inducible protein p78 (mouse)	−1.75	−1.81^*^	−1.14	1.97	−2.00^*^
JAK2	Janus kinase 2	−2.16	1.62^*^	1.82^*^	1.57	1.57^*^
TLR15	Toll-like receptor 15	−1.29	1.95^*^	1.57	1.76^*^	1.84^*^

*Positive and negative numbers indicate upregulation and downregulation respectively. Red indicates fold regulation ≥2, and blue indicates fold regulation ≤ −2*.

* *Indicates fold change values that are significantly different compared to control (P < 0.05)*.

## Discussion

The use of plant extracts in human and animal feeding has been the subject of research due to their broad range of phytochemical compounds (16, 30). Thus, this study examines the effects of organic cranberry and wild blueberry pomaces and their ethanolic extracts in feed on performance, gut microbial community, blood metabolite profile, and spleen immune gene expressions in broiler chicken.

The growth performance data showed that feeding birds with COH300 improved the BW and FE in the early age (day 10); however, BOH300 feed supplementation induced BW improvement at the grower period (days 10–20), while COH150 in feed improved the cumulative FE. Overall, the performance data showed random variation and little consistency without any evidence of dose responses, which is supported by previous research (15, 17, 31). It has been reported that dietary grape pomace did not influence the growth performance at a higher inclusion rate (6%); however, the FE was improved at a lower inclusion rate (3%) (32). In the present study, ethanolic extracts of cranberry pomaces showed a significant improvement of FE compared to control. The reason for a lower BW observed in birds fed pomaces compared to their ethanolic extracts needs to be explored. The presence of a pure form of polyphenols in ethanolic extracts of berry pomaces compared to their pomace could be the reason for higher BW in chicken (13, 14, 33).

Blood serum enzymes such as ALT and AST, produced mainly by the liver, can be indicators of liver disease and the overall health, particularly for obesity and other metabolic syndromes (34). Moreover, there is an increase of lipid metabolism genes associated with the development of Wooden Breast (WB) disease in broiler chickens at 3 weeks of age (35). In our study, blueberry by-products showed a significant treatment effect in lowering ALT and LIP serum concentrations during the grower phase (day 21) of broilers probably due to their compositions. The major anthocyanins detected in the lowbush blueberry pomaces and ethanol extracts used in this study were peonidin 3-glucoside, malvidin 3-galactoside, malvidin 3-glucoside, and cyanidin 3-arabinoside (13). Lowbush blueberry has been reported to contain up to 332 mg/100 g fresh weight (FW) of total procyanidins (36). Sugiyama et al. (37) found that oligomeric procyanidins in apple pomace could be involved in the inhibition of LIP in mice and humans (37). A higher AST:ALT ratio and significantly lower concentrations of AST and ALT were found in the serum of fat birds (34). In the present study, higher AST:ALT ratios were also observed with the blueberry treatments; however, other biomarkers (HDLC) did not change significantly. Hence, further investigation is warranted to elucidate the effect of blueberry by-products on liver enzymes and the metabolism of fat. The low level of TRIG and NEFA observed in birds fed cranberry by-products indicated a decrease of fat deposition. More than 75% of the cranberry flavonols consist of quercetin (38) and have been associated with protection against cardiometabolic risk, such as lowering TRIG both in animal models and in humans (39). A possible mechanism proposed was that quercetin decreased the activity of microsomal TRIG transfer protein (MTP), resulting in the inhibition of intestinal apoB secretion (40). Moreover, proanthocyanidin was also reported to induce hypolipidemia by reducing TRIG in weaned pigs (41). Calcium and phosphorus are essential nutrients involved in many biological processes, and the studied wild blueberry pomaces have been reported to contain at least five times more Ca than in the used cranberry pomaces; however, both pomaces presented similar P content (13). Deficiencies, excesses, or imbalances in Ca and P can result in changes, including an increase or decrease in their absorption from the intestinal lumen. Magnesium has been reported to have several biological functions including muscle and bone growth and antioxidant properties; however, there are limited studies about its role in broilers. The actions of Mg seem to be linked to Ca and P; thus, the right inclusion rate of these minerals in diets can be important in poultry nutrition (42). The decrease of Ca, P, and Mg in the blood of birds fed the highest dose of cranberry pomace deserves more investigation to understand the mechanisms of modulation of these minerals by berry pomaces. In healthy birds, ALB represents the largest part of the protein fractions and reflects the nutrition status and immune system of chicken. While low ALB levels indicated a poor nutrition status, high GLO fractions can be related to a chronic inflammation (43). The reduced AGR in birds fed BACI and cranberry ethanolic extracts may indicate hypoproteinemia and acute or chronic inflammatory processes due to the elevation of GLO. Presently, long-term intense selection for improved BW, FE, and growth rate in broiler chickens result in higher abdominal fat deposition and metabolic changes that may impact the carcass quality. Data generated in the present study show that feeding berry products seems to influence lipid metabolism and serum enzyme secretion in broiler chickens. Necropsy revealed that none of the berry treatments significantly affects the appearance and weight of livers, indicating no liver function deficiency or fat deposition. In general, necropsy data suggested that dietary berry products did not affect the health status of the birds to any large extent.

QA is widely distributed in fruits including cranberry, blueberry, and lingonberry. After absorption from the intestinal tract to the serum, QA is converted into hippuric acid (an antimicrobial compound) or excreted unchanged in urine (44). QA has been found to be an antioxidant agent and an inhibitor of virulence factors of some pathogens such as *Streptococcus, Prevotella*, and *E. coli* (44–46). In the present study, feeding with cranberry or wild blueberry pomaces significantly increased the QA level in the plasma of chickens. Thus, feed supplementation with cranberry and blueberry products could reduce oxidative stresses and improved metabolic functions against reactive oxygen species damage in chickens due to synergistic effects of multiple-phytochemical combinations of both berries (47, 48).

In broiler chickens, it is known that preserving the gut health, which can be influenced by several factors including feeding practices, is important for bird growth performance and overall health. Dietary supplementation of CP1 and BP1 showed significant low NE incidences and lower colony counts compared to the BACI and control treatments. These results indicated that berry pomaces improve the gut heath of broilers by decreasing *C. perfringens* pathogenesis. Antibiotics appear to affect the gut microbiota by reducing the overall diversity, for example, reducing *Lactobacillus* and promoting *Clostridia* in the ilea (3). The microbial population varied in different sites as well as at different raising phases in broilers (49). In the present study, samples from day 21 collection were chosen for analysis based on the importance of this time point during birds' growth (vulnerable to infections), and on at this day, both ceca and cloacae showed similar predominances and abundances of *Firmicutes*. At the phylum level, *Firmicutes, Bacteroidetes*, and *Proteobacteria* were the core microbes in both sites, which is consistent with other studies (50, 51). An increased *Firmicutes*:*Bacteroidetes* (F:B) ratio has been considered as an indicator of obesity due to the improved energy harvesting capacity of *Firmicutes* species (52). Except COH150 treatment, berry pomace treatments reduced the F:B ratio compared to BACI and control; however, no consistent effect of F:B ratio on BW has been observed at 21-day-old broilers. Polyphenols in feed may increase the numbers of several bacteria, including *Bacteroidetes*, which tended to be higher with blueberry by-products in both ceca and cloacae as previously studied (15). These bacteria play an important role in breaking down complex carbohydrates to simpler compounds by encoding enzymes like polysaccharide lyases and glycoside hydrolases (41). BACI treatment resulted in lower abundances of *Proteobacteria* compared to berry pomace treatments, which correlates with an increased population of *Firmicutes* and *Actinobacteria* and probably higher BW*. Actinobacteria* represent a small percentage of the gut microbiota; however, it has been able to maintain gut homeostasis (53). *Eggerthella lenta*, belonging to this phylum in the *Coriobacteriaceae* family, was abundant in the ceca of control birds and those treated with BACI and cranberry products (pomace and ethanolic extracts). However, feed supplementation with blueberry products (pomace and ethanolic extracts) significantly decreased the abundance of this species in the birds' ceca. *Coriobacteriaceae* have been found to be involved in the conversion of bile salts and steroids as well as the activation of dietary polyphenols (54). The tendency of cranberry products to maintain such bacteria compared to blueberry might be explained by the differences in their respective phenolic compounds (13, 55).

Berry pomace has a low pH and is composed of carbohydrates, proteins, lipids, and minerals with a high level of several phenolic compounds (flavonoids, anthocyanins, flavonols). In blueberries, anthocyanins are responsible for their 84% of the total antioxidant capacity, whereas quercetin and ellagic acids are the major flavonoids and total phenolic compounds of cranberries, respectively (55). It has been found that 95% of the total polyphenol intake may be accumulated in the colon and transformed by commensal bacteria into beneficial bioactive compounds (56). In the present experiment, birds fed berry pomace extracts exhibited increased cecal population of potential beneficial bacteria, such as *Enterococcus* and *Lactobacillus* (8.0% with COH300 and 10.9% with CP2, respectively—these counts were lower than 2% in the control). Similar beneficial effects of both berry pomace extracts were observed in the cloaca as well. These beneficial bacteria possess β-glucosidase activity and have the ability to metabolize berry anthocyanins into phenolic metabolites like *p*-couramic acid and benzoic acid (57). On the other hand, polyphenols in the berry pomaces may act as prebiotic support for growth of these beneficial bacteria, which produce lactate as the main fermentation product that can be assimilated in the cecum, serving as an energy source (58). Carbohydrates of berry pomaces could also stimulate the growth of these beneficial bacteria, which catabolize glycan, leading to the secretion of acetate, lactate, formate, and butyrate (59). Besides, iron-chelating activities of pomace compounds such as tannin could induce iron-poor conditions, which are favorable to *Lactobacillus*, as these bacteria do not require iron for growth (60). Accordingly, data of the present study showed an increase of butyrate-producing genera such as *Ruminococcus* and *Coprococcus* in cranberry pomace–fed broiler ceca similar to what was observed in broiler chickens fed chlortetracycline, virginiamycin, and amoxicillin prophylactically for growth promotion (61). The above changes induced by tested products in this study could explain, at least in part, the low prevalence of subclinical NE caused by *C. perfringens* and coccidiosis due to *Eimeria* species. These data indicate that berry pomaces could be developed as alternatives to traditional antibiotics in broiler production.

Overall, in all treatments, *Firmicutes* and *Actinobacteria* were negatively correlated with mortality, whereas *Bacteroidete* and *Cyanobacteria* were positively correlated with it. Increased cloacal *Firmicutes* facilitates nutrient absorption, whereas the opposite scenario has been observed with *Bacteroidetes* (62). In our cloacal samples, we found increased *Firmicutes* vs. *Bacteroidetes* with BACI, COH150, and COH300 feed treatments (*P* > 0.05), which may improve the nutrient absorption by the gut microbiota and resulted in a lower FE. However, we did not see a significant correlation between *Firmicutes* and FE. Rather, *Firmicutes* were negatively related in lowering some of the important blood metabolites like CHO, NEFA, and TRIG. Thus, feed supplementation by the cranberry products in broilers could improve production efficiency similar to BACI. Conversely, *Bacteroidetes* help to promote intestinal digestion, nutrient utilization, and hind gut fermentation of substrates to produce SCFA, as well as promoting the conversion of the absorbed SCFA to more complex compounds in the liver (63). We found that blueberry by-products significantly increased the abundances of *Bacteroidetes* more than any other treatments, particularly in the ceca samples, presumably related to the increase of BW during the growing period and to the reduction of blood serum enzymes like ALT and LIP.

High production performance can be harmful to immunity and intestinal integrity in broiler chickens. In the present study the prevalence of subclinical intestinal NE lesions due to *C. perfringens* was significantly low in birds fed cranberry pomace, along with high relative abundances of *Eggerthella, Ruminococcus*, and *Lactobacillus* in the gut. Moreover, cranberry pomace treatment significantly increased the QA level and influenced the lipid metabolism by reducing the level of TRIG and NEFA in blood. Gut microbiota play an important role in shaping immunity by influencing the balance between pro-inflammatory and immune regulatory responses to maintain immune homeostasis (64). The above observed biological activities with cranberry by-products led us to investigate its effects on broilers' spleen immunity. The spleen is a secondary lymphoid organ for both innate and adaptive immune response in chickens and therefore, its gene expression is commonly used as an indicator of immune response (65–67). Cranberry product treatment influenced the expression of genes encoding CD14, involved in innate immunity, and IL4, IL5, and CSF2, involved in adaptive immunity. These gene modulation effects could be related to effects on the enrichment of beneficial bacterial populations such as *Eggerthella* and *Lactobacillus* in the gut and accumulation of QA in the blood. Probiotic bacteria like *Lactobacillus* spp. were reported to reduce the production of pro-inflammatory cytokines like IL12 (64). The present study indicated that dietary cranberry products could reduce intestinal inflammation, while maintaining the intestinal homeostasis in broilers. Future investigations are warranted to establish the mechanisms involved in these processes.

Broiler production in Canada and in the United States of America is facing constraints. In fact, the consequences of broiler production for environmental, food safety, and animal welfare issues are forming the opinions of consumers, who are now demanding organic or antibiotic-free poultry products. Gut microbiota has been associated with wellness and diseases. Thus, understanding the molecular mechanisms by which these effects occur in the host will be useful in designing strategies to modulate the gut bacterial composition. The present study showed that feeding with cranberry and wild blueberry products influenced lipid metabolism, mineral profile, and gut microbiota in broiler chickens. However, for most of the estimated parameters, no evidence of a dose-dependent response was noted. On some measured parameters, pomaces at 1% in feed seemed to be a more effective dose than 2%, which suggested a possible concentration-dependent response threshold. Phenolic-enriched extractives from cranberry pomace appeared to be the most effective products on FE. Therefore, more research on berry products would help in designing strategies to reduce the use of antibiotics and lessen antibiotic resistance in broilers.

## Data Availability Statement

The raw sequence read of bacterial 16S rRNA genes of the 140 (70 cecal and 70 cloacal) samples obtained in this study has been submitted to the Sequence Read Archive (SRA) database of the National Center for Biotechnology Information as FASTQ files under study accession number PRJNA273513.

## Ethics Statement

The animal study was reviewed and approved (protocol # 16-AV-314) by the Animal Care Committee of the Center de recherche en sciences animales de Deschambault (CRSAD, Deschambault, QC, Canada) according to guidelines described by the Canadian Council on Animal Care.

## Author Contributions

MD and QD conceived and designed the experiments. DL, QD, XY, MI, HL, LM, JT, and MD performed the experiments and data analysis. MD, KR, YK, and HY contributed reagents and materials. QD and MD wrote the paper. MD, KW, MM, DL, JT, and MI reviewed and edited the manuscript. All authors read and approved the final manuscript.

## Conflict of Interest

The authors declare that the research was conducted in the absence of any commercial or financial relationships that could be construed as a potential conflict of interest.
