# Corrigendum: Gut Microbiota, Blood Metabolites, and Spleen Immunity in Broiler Chickens Fed Berry Pomaces and Phenolic-Enriched Extractives

**DOI:** 10.3389/fvets.2020.00541

**Published:** 2020-09-04

**Authors:** Quail Das, Md. Rashedul Islam, Dion Lepp, Joshua Tang, Xianhua Yin, Lili Mats, Huaizhi Liu, Kelly Ross, Yan Martel Kennes, Hassina Yacini, Keith Warriner, Massimo F. Marcone, Moussa S. Diarra

**Affiliations:** ^1^Department of Food Science, University of Guelph, Guelph, ON, Canada; ^2^Guelph Research and Development Centre, Agriculture and Agri-Food Canada, Guelph, ON, Canada; ^3^Summerland Research and Development Centre, Agriculture and Agri-Food Canada, Summerland, BC, Canada; ^4^Centre de Recherche en Sciences Animales de Deschambault, Deschambault, QC, Canada

**Keywords:** broilers, cranberry and blueberry pomaces, blood metabolites, gut microbiota, spleen, immunity

In the published article there was an error in the Copyright statement. As the authors are employees of the Canadian Government, copyright is held with the Government. The correct statement appears below. The authors apologize for this error and state that this does not change the scientific conclusions of the article in any way. The original article has been updated.

## Copyright Statement

Copyright © 2020 Her Majesty the Queen in Right of Canada, as represented by the Minister of Agriculture and Agri-Food Canada. This is an open-access article distributed under the terms of the Creative Commons Attribution License (CC BY). The use, distribution or reproduction in other forums is permitted, provided the original author(s) and the copyright owner(s) are credited and that the original publication in this journal is cited, in accordance with accepted academic practice. No use, distribution or reproduction is permitted which does not comply with these terms.

